# Cross-cultural adaptation and validation to Brazilian Portuguese of two measuring adherence instruments for patients with type 1 diabetes

**DOI:** 10.1186/1758-5996-6-141

**Published:** 2014-12-16

**Authors:** Gabriela Heiden Teló, Martina Schaan de Souza, Beatriz D’Agord Schaan

**Affiliations:** Post-graduate Program, Endocrinology, Universidade Federal do Rio Grande do Sul, Porto Alegre, RS Brazil; Universidade Federal do Rio Grande do Sul, Porto Alegre, RS Brazil; Endocrine Division, Hospital de Clínicas de Porto Alegre, Porto Alegre, RS Brazil; Serviço de Endocrinologia- Hospital de Clínicas de Porto Alegre, Rua Ramiro Barcelos 2350, prédio 12, 4 andar, 90035-003 Porto Alegre, RS Brasil

**Keywords:** Type 1 diabetes, Adherence, Compliance, Treatment

## Abstract

**Background:**

The purpose of this study was to carry out a cross-cultural adaptation to Brazilian Portuguese, validation, and comparison of two questionnaires to measure adherence in patients with type 1 diabetes. There are no validated instruments to measure treatment adherence in Brazilian patients with type 1 diabetes.

**Methods:**

Type 1 diabetes outpatients of a tertiary hospital in Southern Brazil were recruited to examine psychometric properties of the Diabetes Self-Management Profile (DSMP) and Self-Care Inventory-revised (SCI-R) adapted to Brazilian Portuguese. Analyses assessed the reliability and validity according to its associations with glycated hemoglobin (A1C). Seventy-five patients [age: 34.9 ± 13.7 years; A1C: 9.2 ± 2% (75 mmol/mol); diabetes duration: 18.1 ± 11.8 years] were evaluated.

**Results:**

The translated versions of the instruments showed adequate internal consistency (DSMP Cronbach’s α =0.76; SCI-R Cronbach’s α =0.71). A positive correlation was found between all the items and total scores, except for item 12 in DSMP and item 13 in SCI-R, and for this reason, these items were excluded from the translated versions. In predictive validity analysis, A1C correlated significantly with the DSMP total (*r* = −0.46) and with the SCI-R total (*r* = −0.44).

**Conclusions:**

The Brazilian Portuguese versions of DSMP and SCI-R yielded a reliable and valid tool to measure adherence treatment for patients with type 1 diabetes, with a significant correlation between total scores and A1C.

## Background

Brazil is the fifth largest country in the world, with an estimated population of 191 million [1]. Recent data suggests that 2.5 million Brazilians live abroad, of which, over one million in the United States of America [2]. Diabetes affects 8.3% of the population, which contributes to why it is one of the leading causes of premature illness and mortality [3]. The global variation in the incidence of type 1 diabetes is known to be high [4, 5]. In Brazil, the estimated incidence of type 1 diabetes (27.20/100,000 per year) is considered very high and is increasing, and a wide variation is observed between and within ethnic groups, with higher incidence in Caucasians [6] (54% of the Brazilian population) [7].

Major clinical trials of type 1 diabetes have demonstrated the benefits of intensive glycemic control in preventing chronic diabetes complications [8, 9]. To prevent serious morbidity and mortality, diabetes treatment requires dedication to demanding self-care behaviors in multiple domains, including diet plan, physical activity, medications, glucose monitoring and symptom management [10]. Among strategies to achieve glycemic targets, improving adherence to diabetes-related tasks is very important, as it predicts glycemic control and healthy outcomes [9, 11]. Published estimates of non-adherence rates have ranged from 40% to 90% across studies and measures, contributing to poor glucose control [12, 13]. A recent nationwide multicenter study in Brazil reported that only 13.2% of patients with type 1 diabetes were at the goal of A1C [14] recommended by the American Diabetes Association [15]. This finding was associated with lower economic status; however, no data were available about adherence to diabetes management [14, 16]. At a public hospital in Southern Brazil, non-adherence was the most common precipitating factor of diabetic ketoacidosis, responsible for 49% of this emergency hospitalization [17]. Moreover, medication noncompliance was associated with 41% higher inpatient costs [18] and a better adherence was found to be associated with improved glycemic control and decreased health care resource utilization [10], resulting in an annual estimated cost savings of $1 billion [18].

Despite the well-known importance of adherence in diabetes treatments and tasks in order to achieve expected goals to prevent chronic complications, there are few easy-to-use instruments with established psychometric properties to assess it. The Diabetes Self-Management Profile (DSMP) is a validated, semi-structured interview that extensively measures adherence to type 1 diabetes management tasks over the previous 3 months [19]. It requires 30–40 min to be administered, and because of the long duration its use in clinical care may not be practical. A concise version was developed [20], but with decreased internal consistency. The Self-Care Inventory-revised (SCI-R) is a brief, psychometrically sound measure of perceptions of adherence to recommended diabetes self-care behaviors of patients with diabetes [21], but more superficially explores each item related to non-adherence.

There is no instrument to measure adherence to the treatment of type 1 diabetes adapted to Brazil. The aim of this study was to adapt to Brazilian Portuguese, validate, and compare the two main available instruments. One of them is easy to use in clinical practice and the other is more complete with useful information for clinical research purposes.

## Methods

### Participants

This study was carried out in a diabetes outpatient clinic of a university hospital in Brazil from June to December 2012. Eligibility criteria included: ages 11 and older [19]; previous diagnosis of type 1 diabetes; disease duration of at least 12 months; and Brazilian nationality. The exclusion criteria were a development disability or a psychiatric disorder that would be an obstacle in completing the structured interview. Patients were searched for in the hospital’s medical record database, and those who met these criteria were given study information. Interested eligible patients or their legal guardians signed an informed consent form, conforming to the Helsinki Declaration of 1975, as revised in 2000 and 2008. This study was previously approved by the Institutional Ethics Committee.

### Instruments

The authors of the original questionnaires were asked permission by email, and the study began only after their authorization. In this study, two instruments were randomly applied by two interviewers, both well-advised of the research objectives and trained in order to maintain a neutral position while the patients filled out the questionnaires.

The DSMP, originally validated by Harris et al., is a structured interview developed to assess self-management among young patients with type 1 diabetes. It consists of 25 items measuring 5 domains: exercise, hypoglycemia, diet, blood glucose test, and insulin dose. This instrument has revealed adequate internal consistency, inter-rater agreement and test-retest reliability, in addition to a correlation with glucose control, measured by A1C [19]. These results were maintained in cross-cultural adaptations thereafter [22].

The SCI-R, validated by La Greca et al., is a 15-item questionnaire, on a 5-point Likert scale that reflects how well the subjects followed recommendations for self-care during the past month (i.e., 1 = “never” to 5 = “always”). The measure requires 10–15 min to be applied, and has acceptable internal reliability and correlation between glycemic control and total score [21]. In both instruments higher scores indicate more meticulous self-management.

After the questionnaires were administered, researchers interviewed the patients to obtain information about clinical and demographic data. Economic status was defined in accordance with the Brazilian Economic Classification Criteria [23]. A1C (high-performance liquid chromatography method) was obtained from the patients records over the past 3 months and if not available, from a new blood sample.

### Procedures and measures

The initial translation of the original instruments into Brazilian Portuguese was performed by two independent translators who were native speakers of Brazilian Portuguese, were fluent in both languages and had different professional profiles from the researchers. One was aware of the underlying objectives of the instruments to be translated and the concepts involved in order to offer a more reliable restitution of the intended measurement. Resulting synthesis versions were obtained, and the words and phrases that presented differences were consensually readapted and defined by the translators [24].

The synthesis version of the instruments was back-translated by two independent translators, who were native speakers of English as well as fluent in both languages. Neither of them were aware of the underlying objectives of the instruments [24, 25].

Based on the translations and back-translations obtained, a committee was assembled, in a face-to-face meeting, in order to produce a final version of the modified tool. This committee was composed of two endocrinologists (including the principal investigator), the four translators involved, all bilinguals and one of them a specialist in Linguistics. The committee carefully considered each of the items of the instruments in order to better adapt them, with the goal of ensuring that the translation is fully comprehensible and verifying cross-cultural equivalence of source and final version, considering semantic, idiomatic, experiential and conceptual equivalence [25].

The final stage of adaptation process was the pretest of the new questionnaires, applied in 40 previously selected patients [24]. The subjects completed the instruments and were interviewed to find out what they thought was meant by each questionnaire item and their chosen response [24, 25]. The cognitive debriefing was used to generate a final version of the translated instruments, based on participants’ feedback, to confirm the acceptability of the translation. Test-retest reliability over 3 months was determined in order to stabilize the sample and to check the intra-observer variability [19, 24]. Upon completion of this cross-cultural adaptation, in order to verify that the instruments lead to valid conclusions, analyses were conducted on 75 patients (including the 40 initially evaluated) [26] to assess the construct and discriminant validity [24, 25]. All patients answered the same questions with a second interviewer, within 1 to 2 weeks of the previous evaluation, in order to evaluate the inter-rater agreement [19].

### Data analysis

Data are presented as mean ± standard deviation (SD) or percentage, and median and interquartile range (25 and 75 percentiles). Statistical analyses employed SPSS 19.0 and the level of significance was defined as α =0.05. T-test and Mann–Whitney were used to analyze quantitative data, and Chi-square test was used for categorical variables.

Cronbach’s α was calculated to measure internal consistency, and item-total correlations were computed. In this analysis, values above 0.7 were considered acceptable [25]. In addition, we analyzed the impact of removal of each question on the value of Cronbach’s α, which could lead to the exclusion of a particular item. A confirmatory factor analysis was used to evaluate it. Test-retest and inter-rater agreement were measured by intraclass correlation coefficient.

Pearson correlation coefficient was used to examine predictive validity between the instruments total scores and A1C, and convergent validity between DSMP and SCI-R scores. Dichotomization of the scores into poor [A1C ≥8% (≥64 mmol/mol)] and good glucose control [A1C <8% (<64 mmol/mol)] [27] was performed to evaluate the best cutoff to discriminate it, using receiver operating characteristic (ROC) curve.

## Results

### Descriptive statistics

The initial search of medical records included 123 patients, from which 44 declined to participate, 3 were excluded because of developmental disabilities and another was excluded for not returning in subsequent reevaluation. The sample consisted of 75 subjects 34.9 ± 13.7 years of age, 66% female and 89% Caucasian. Mean participant duration of diabetes was 18.1 ± 11.8 years, and A1C was 9.2 ± 2.0% (77 mmol/mol). Other characteristics are available in Table 1.

**Table 1:** **Clinical and demographic data of the study population**

Variable	N = 75
**Gender, f (%)**	42 (56)
**Age, y**	34.9 ± 13.7
**Duration of diabetes, y**	18.1 ± 11.8
**Ethnicity, (%)**	
Caucasian	67 (89.3)
Economic status*, (%)	
A1	0 (0)
A2	0 (0)
B1	0 (0)
B2	9 (12)
C1	39 (52)
C2	24 (32)
D	3 (4)
E	0 (0)
**Years of study**	10.7 ± 4.0
**A1C, % (mmol/mol)**	9.2 ± 2.0 (77)

Data presented as number (%), mean ± SD, median and 25% and 75% quartiles; f = female, y = years.

*High (A1, A2, B1), medium (B2, C1), low (C2) and very low (D, E).

### Reliability

The original Brazilian Portuguese version of the DSMP was composed of 25 items, and could be completed in approximately 20 min. Item-total correlations were calculated separately for each item in the Brazilian Portuguese version of the questionnaires. All item-total correlations, except one, were in the expected direction, ranging from 0.01 to 0.76. Confirmatory factor analysis was conducted to examine an “a priori” five-factor structure, which consisted of each subscale identified as a factor. The same item did not load onto the factor for this version and was eliminated [In the past 3 months, how often have you eaten less than what is recommended for your meal plan?], which increased Cronbach’s α from 0.74 to 0.76. Internal consistency (Cronbach’s α) of the DSMP subscales was all <0.7, indicating that the subscales are not reliable when used separately. Correlations between the total and subscale scores were as follows: exercise, *r* = 0.27 (*P* = 0.02); hypoglycemia, *r* = 0.65 (*P* <0.01); diet, *r* = 0.72 (*P* <0.01); blood glucose test, *r* = 0.88 (*P* <0.01); and insulin dose, *r* = 0.62 (*P* <0.01). On the SCI-R, Cronbach’s α was initially 0.69, with all item-total correlations in the expected direction, ranging from 0.01 to 0.65. The removal of one item [Wear a medic alert ID] led to a significant increase in internal consistency, therefore it was excluded. The final Cronbach’s α was 0.71 (Table 2). The application of this 14-question translated version took less than 10 min.

**Table 2:** **Technical features, psychometric properties and predictive validity of the Brazilian versions of the DSMP and SCI-R (N = 75)**

	DSMP	SCI-R
**Items (n)**	24	14
**Application time (min)**	20-30	8-10
**Mean total score**	41.4 ± 10.6	47.8 ± 8.1
**Cronbach’s α**	0.76	0.71
**Correlation with A1C (r)**	−0.46	−0.44
**Cutoff value for non-adherence**	≤ 41	≤ 48

Data presented as number (%), mean ± SD; *r* = Pearson correlation coefficient. DSMP: Diabetes Self-Management Profile; SCI-R: Self-Care Inventory-revised.

Inter-rater agreement was established through two independent interviews of all participants and by calculating intraclass correlation coefficient. Inter-rater agreement was 0.91 on the DSMP and 0.92 on the SCI-R.

Test-retest reliability over 3 months was determined using data from the first 40 patients. The intraclass correlation coefficient between the baseline and 3-month scores on the DSMP was 0.99 (exercises, 0.99; hypoglycemia, 0.99; diet, 0.60; blood glucose test, 0.99; insulin dose, 0.97) and on the SCI-R it was 0.99.

### Validity

Predictive validity was determined by correlating DSMP and SCI-R scores with A1C results. A1C correlated significantly with the DSMP total (*r* = −0.46, *P* <0.01), as well as with 3 of the 5 subscales (diet, *r* = −0.42; blood glucose test, *r* = 0.40; insulin dose, *r* = 0.40, *P* <0.01) and with the total SCI-R (*r* = −0.44, *P* <0.01).

In the analysis of convergent validity, a strong correlation (*r* = 0.88, *P* <0.01) was found between the DSMP and the SCI-R scores.

Mean DSMP total scores were 41.4 ± 10.6, and the best cutoff value for classifying a patient as having a higher or lower adherence score, considering A1C results, was 41 (area under the ROC-curve = 0.73, *P* <0.01), where higher scores indicate greater adherence. The mean of SCI-R total scores was 47.8 ± 8.1, in which the best cutoff value for classifying a patient as having a higher or lower adherence score was 48 (area under ROC-curve = 0.71, *P* <0.01), where higher scores also indicate greater adherence [27]. There were no significant clinical or demographic differences between both groups based on these classifications (Table 3).

**Table 3:** Clinical and demographic differences between patients with different adherence profiles* (N =75)

Variable	DSMP		P	SCI-R		P
	Lower adherence score	Higher adherence score		Lower adherence score	Higher adherence score	
**N (%)**	36 (48)	39 (52)		33 (44)	42 (56)	
**Gender, f**	21 (58.3)	21 (53.8)	0.67	20 (60.6)	22 (52.4)	0.50
**Age, y**	33.7 ± 13.3	35.0 ± 14.1	0.91	32.6 ± 12.0	36.7 ± 14.8	0.15
**Duration of diabetes, y**	16.7 ± 13.7	19.4 ± 9.8	0.09	15.6 ± 11.3	20.1 ± 11.9	0.06
**Caucasian**	30 (83.3)	37 (94.9)	0.09	29 (87.9)	38 (90.5)	0.34
**Years of study**	10.0 ± 3.6	11.4 ± 4.1	0.94	9.4 ± 3.72	11.7 ± 3.8	0.60
**Diabetes complications**	14 (38.9)	14 (35.9)	0.97	13 (39.4)	15 (35.7)	0.86
**BMI, kg/m** ^**2**^	24.2 ± 3.6	25.1 ± 4.0	0.44	24.1 ± 3.3	25.0 ± 4.1	0.64

Data presented as number (%), mean ± SD, median and 25% and 75% quartiles; f = female, y = years, BMI = body mass index. DSMP: Diabetes Self-Management Profile; SCI-R: Self-Care Inventory-revised.

*Based on best cutoff for classifying a patient as having a higher adherence score (≥41 for DSMP and ≥48 for SCI-R).

## Discussion

The results of the present study provided support for the validity of a Brazilian Portuguese version of two measuring adherence instruments for patients with type 1 diabetes, DSMP and SCI-R, indicating that they could be used reliably in future studies.

The reliability of both instruments was evaluated using several methods. Cronbach’s α was acceptable for both and similar to the original version of the DSMP [19], suggesting that, despite modifications, the scales remained internally consistent. A better value was found in the original SCI-R (Cronbach’s α = 0.87), however it also included patients with type 2 diabetes [21]. Furthermore, agreement was found between the two independent raters in total scores as well as in the five subscales of DSMP. The test-retest reliability over 3 months was higher than 0.9 in total scores and in all of the subscales of DSMP, with the exception of one, diet, a finding we attributed to long period between assessments and seasonal dietary variations [28] considering that this study started in the winter and finished in the summer. In both instruments, we excluded one item due to lack of benefit as well as a significant improvement in reliability after their exclusion. In the United Kingdom version of SCI-R, the same item was excluded [29].

Diabetes Self-Management Profile and SCI-R scores obtained from the original instruments correlated significantly with A1C in the expected direction, underscoring the importance of evaluating adherence in these patients, as it is correlated with glucose control [11]. It demonstrated that more careful self-management of diabetes was associated with better glycemic control. This study found a stronger correlation between total scores and A1C than the original instruments (*r* = −0.28 for DSMP and *r* = −0.37 for SCI-R) [19, 21], which could be related to having the same time period between A1C sample and questionnaire application, a reliable method for measuring A1C [15], and extensive interviewer training.

High concordance with the original versions of both instruments was found in the translated measures in this study. Forward and back-translations were used to maintain semantic and conceptual equivalence, allowing better analysis of adherence in Brazilian patients with type 1 diabetes. Mean SCI-R score in Brazil was much lower than that of the English version (65 ± 15) [21], as well as the mean DSMP score (58.5 ± 9.1). This finding is consistent with the results presented by Gomes et al. [14], who examined the prevalence of Brazilian adults with type 1 diabetes that met the goals of care in daily clinical practice, and found that only 13.2% achieve them. Considering the fact that the treatment guidelines of the Brazilian Diabetes Society [30] are essentially the same as those of the American Diabetes Association [15], other factors such as, noncompliance, low educational level and poor economic income might interfere with diabetes care in Brazil.

Using the available validated instruments, we evaluated their possible ability to discriminate between patients with a better or worse adherence profile, dichotomizing them into poor and good controls [27]. We found statistically significant cutoffs, which may be useful in the interpretation of the scores during the application of the instruments in clinical or research settings. The results point out that 41 and 48 were the best cutoff points for DSMP and SCI-R scores, respectively.

There are a number of limitations in this study. First, the Brazilian Portuguese version of DSMP was adapted from the original English version, however it included two versions, one for the patients and one for their parents [19]. We decided to adapt only the patient version, aiming to obtain information directly from patients about their diabetes care. Moreover, although our sample was older than the sample in the English DSMP publication, patients with the same age range were included. The reason for this is because our institution predominantly provides diabetes care for adult patients. Nevertheless, the results we encountered were similar to those found in the original version of the DSMP. Regarding SCI-R, it primarily evaluates patients with type 1 and type 2 diabetes, including youth and adults [21]. As our study intended to assess compliance of patients with type 1 only, we did not include patients with type 2 diabetes, and it may have led to different results when compared to the original version. In addition, our sample size was calculated based on literature recommendations for studies with this methodology [26], and not based on possible differences between patients with better and worse adherence profile. This may be the reason why we did not find statistically significant clinical or demographic differences between patients with high and low adherence scores. On the other hand, in a meta-analysis that evaluated adherence and glycemic control in pediatric type 1 diabetes patients, no socio-demographic or disease characteristics were associated with noncompliance, suggesting that all patients should experience better glycemic outcomes with adherence promotion [11]. Finally, we verified the sensitivity of the instruments to detect differences between groups. In the absence of a gold standard, we opted to use A1C as a parameter to differentiate the groups, considering its importance as an indicator of metabolic control [15] and the high correlation reported between metabolic control and adherence [11]. Nevertheless, the correct methodology implemented allows us to validate the results.

## Conclusion

In conclusion, the Brazilian Portuguese adapted versions of the evaluated instruments demonstrated acceptable psychometric properties and validity with similar results. Both of them can provide an important alternative to assist researchers in the assessment and interpretation of treatment adherence in patients with type 1 diabetes. However, considering the similar results between both surveys and the shorter time for administration of the SCI-R, this questionnaire could be a better option in the clinic right away.
